# Final analysis of colorectal cancer patients treated with irinotecan and 5-fluorouracil plus folinic acid neoadjuvant chemotherapy for unresectable liver metastases

**DOI:** 10.1038/sj.bjc.6603988

**Published:** 2007-09-25

**Authors:** C Barone, G Nuzzo, A Cassano, M Basso, G Schinzari, F Giuliante, E D'Argento, N Trigila, A Astone, C Pozzo

**Affiliations:** 1 Unit of Medical Oncology, Department of Internal Medicine, Catholic University of Sacred Heart, Rome, Italy; 2 Unit of Hepatobiliary Surgery, Department of General Surgery, Catholic University of Sacred Heart, Rome, Italy

**Keywords:** colorectal cancer, irinotecan, FOLFIRI, liver metastases, neoadjuvant

## Abstract

We have previously reported that neoadjuvant therapy with modified FOLFIRI enabled nearly a third of patients with metastatic colorectal cancer (mCRC) to undergo surgical resection of liver metastases. Here, we present data from the long-term follow-up of these patients. Forty patients received modified FOLFIRI: irinotecan 180 mg m^−2^, day 1; folinic acid, 200 mg m^−2^; and 5-fluorouracil: as a 400 mg m^−2^ bolus, days 1 and 2, and a 48-h continuous infusion 1200 mg m^−2^, from day 1. Treatment was repeated every 2 weeks, with response assessed every six cycles. Resected patients received six further cycles of chemotherapy postoperatively. Nineteen (47.5%) of 40 patients achieved an objective response; 13 (33%) underwent resection. After a median follow-up of 56 months, median survival for all patients was 31.5 months: for non-resected patients, median survival was 24 months and was not reached for resected patients. Median time to progression was 14.3 and 5.2 months for all and non-resected patients, respectively. Median disease-free (DF) survival in resected patients was 52.5 months. At 2 years, all patients were alive (8 DF), and at last follow-up, eight were alive (6 DF). Surgical resection of liver metastases after neoadjuvant treatment with modified FOLFIRI in CRC patients achieved favourable survival times.

Worldwide, colorectal cancer (CRC) is the fourth most common malignancy in men and the third most common in women. In 2002, the disease accounted for more than one million new cancer cases and caused 528 978 deaths (Parkin *et al*, 2005). Approximately 25% of CRC patients present with metastatic disease, with the liver being the most common site of metastasis. Over time, 40–50% of newly diagnosed CRC patients will develop metastatic disease (Saltz *et al*, 2000). The 5-year survival rate for patients with metastatic CRC (mCRC) is 5–30% (Van Cutsem *et al*, 2005).

Patients who are able to undergo resection of isolated liver metastases have the chance of cure or improved survival. If cases are carefully selected for surgery, the 5-year survival rate may be as high as 60% (Fernandez *et al*, 2004; Pawlik *et al*, 2005). However, only 10–20% of patients with mCRC are suitable for liver resection at presentation (Adam, 2003). Over the last 10 years, the introduction of two new active cytotoxic chemotherapy drugs and several targeted biological agents has improved the outlook for patients with mCRC. Median survival times in excess of 20 months can now be expected with the first- and second-line use of irinotecan and/or oxaliplatin in combination with 5-fluorouracil (5-FU)/folinic acid (FA). This compares favourably with the 12–14 months previously achieved with 5-FU/FA alone (Douillard *et al*, 2000; Thirion *et al*, 2004).

These more active chemotherapy regimens are also associated with higher response rates of 39–62% (Bismuth *et al*, 1996; Douillard *et al*, 2000; Saltz *et al*, 2000; Tournigand *et al*, 2004; Alberts *et al*, 2005; Kohne *et al*, 2005). In addition, as many as 68% of responding patients with initially unresectable liver disease have been reported to be subsequently eligible for secondary resection of their metastases (Alberts *et al*, 2005). Indeed, the importance of maximising response rates in this setting was highlighted by the retrospective analysis of Folprecht *et al* (2005), which demonstrated a strong correlation between response and resection rates, especially in selected patients with no disease outside the liver.

The present study previously reported that 32.7% of patients with CRC were able to undergo potentially curative surgical resection of initially unresectable liver metastases following neoadjuvant chemotherapy with an irinotecan/5-FU/FA combination (modified FOLFIRI) (Pozzo *et al*, 2004). This paper reports on the long-term follow-up of these patients, providing data on overall survival, disease-free (DF) survival and time to progression (TTP).

## MATERIALS AND METHODS

### Patients

Patients aged 18–75 years with unresectable colorectal liver metastases were eligible for entry into the study. Other eligibility criteria were no evidence of extrahepatic disease or involvement of more than 70% of the liver; Eastern Cooperative Oncology Group (ECOG) performance status of 2 or less; no previous chemotherapy for advanced disease; any adjuvant chemotherapy completed at least 6 months prior to study entry; no history of other malignancies; adequate bone marrow (WBC >3 × 10^9^ l^−1^, platelets >100 × 10^9^ l^−1^, haemoglobin >10 g dl^−1^), liver (total bilirubin <2 mg dl^−1^, aspartate aminotransferase or alanine aminotransferase <3 × upper limit of normal) and renal function (blood urea nitrogen ⩽30 mg dl^−1^, creatinine clearance >60 ml min^−1^, serum creatinine ⩽1.5 mg dl^−1^); and a life expectancy of more than 3 months. Written informed consent was provided by all patients.

### Chemotherapy

Modified FOLFIRI was administered every 2 weeks and consisted of the following: irinotecan 180 mg m^−2^ given i.v. on day 1; 5-FU 1200 mg m^−2^ continuous i.v. infusion over 48 h from day 1; FA 200 mg m^−2^ i.v. on days 1 and 2 and 5-FU 400 mg m^−2^ i.v. bolus on days 1 and 2. Response was assessed every 12 weeks (six cycles). In cases of neutropaenia (⩽1.5 × 10^9^ l^−1^), thrombocytopaenia (⩽100 × 10^9^ l^−1^) or significant non-haematologic toxicity, treatment was delayed for 1 week. In the event of grade 3/4 neutropaenia or diarrhoea, the doses of irinotecan and 5-FU were reduced by 25%. The dose of 5-FU only was similarly reduced in the event of grade 3/4 stomatitis. Treatment was interrupted for grade 4 haematologic toxicity or grade 4 gastrointestinal toxicity (lasting >1 week after a previous dose reduction or a delay in dose administration of >2 weeks).

Chemotherapy was continued until an objective response was achieved (indicating suitability for surgery), or until disease progression or unacceptable toxicity. Those patients who underwent resection of their liver metastases received six further cycles of the same chemotherapy regimen postoperatively. Patients with progressive disease were treated at the discretion of the investigator.

### Assessments

Initial unresectability of liver metastases was assessed as described previously, using the criteria set out by the institution at which the study was carried out (Pozzo *et al*, 2004).

## RESULTS

### Patients

Forty patients were enrolled into the study over 2.5 years up to January 2003. The patients' baseline characteristics are outlined in Table 1. The median age of the patients was 58.7 years (range: 32–75 years) and the majority were male (60%). All but one patient had an ECOG performance status of 0. The majority of patients (67.5%) had synchronous metastases, with the remainder being metachronous. At study entry, 21 patients (52.5%) had less than three metastases and 11 (27.5%) had more than six. Twelve (30%) patients had at least one lesion ⩾5 cm in diameter and seven (17.5%) patients had metastases that were unfavourably located in the hilum.

Of those patients with liver metastases that were considered to be unresectable, 35% had large numbers of metastases, 25% had large metastases, 5% had inadequate liver reserve and 35% had surgically inaccessible metastases (Table 1).

### Response to chemotherapy

**Figure 1:**
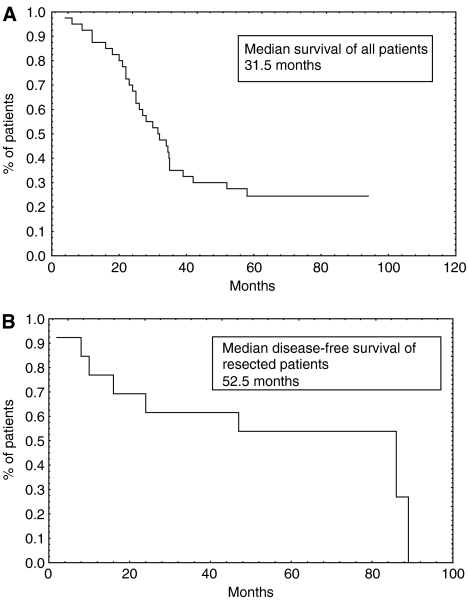
(**A**) Median overall survival. (**B**) Median disease-free survival.

As of December 10 2006, median follow-up was 56 months. All 40 patients had been evaluated for response yielding an objective response rate of 47.5%, including two complete responses. Of the 19 (47.5%) patients who experienced an overall response, 16 were considered to be eligible for surgery (after a median of eight cycles of chemotherapy per patient (range: 6–12)) and 13 underwent resection of their liver metastases. The median overall survival of these patients has not yet been reached. The median survival for all 40 patients was 31.5 months (Figure 1A) and in non-resected patients, 24 months (Table 2). Nine out of 40 patients (22.5% (95% CI: 10.5–34.5%)) were still alive, including one not resected patient. The median TTP for all patients was 14.3 months (range: 2–49), and in non-resected patients, 5.2 months.

### Adverse events

The treatment was well tolerated and the adverse events that were observed and described previously were those typical of the cytotoxic agents used (Pozzo *et al*, 2004). There were no delayed onset adverse events. There were no significant differences in the incidence and severity of adverse events experienced by patients during the additional postresection chemotherapy phase compared with those reported for the initial phase of treatment.

### Surgery

Among the 13 patients who underwent surgery, there were 13 R0 liver resections. Most of these patients (10 out of 13) underwent multiple segmental resections. As reported previously, the resection rate following chemotherapy was highest in those patients with large (>5 cm) metastases (6 out of 10) (Pozzo *et al*, 2004). Also, there was no postoperative mortality in the 2 months following surgery. One patient experienced anaemia as a consequence of postoperative bleeding (Pozzo *et al*, 2004).

### Disease-free survival

The median DF survival of the resected patients was 52.5 months (Figure 1B). At the time of reporting, a median of 56 months from resection, 6 (46% (95% CI: 19.3–72.9%)) of the 13 radically resected patients were still disease free and 8 (62%) of the 13 were still alive (Table 2).

## DISCUSSION

After a median follow-up period of nearly 60 months, the median survival for all patients was 31.5 months. This is one of the highest reported median survival times for a prospective study in similar groups of patients treated with standard combinations of cytotoxic agents. Moreover, currently, the median survival time for those patients who underwent secondary resections of their liver metastases has not yet been reached.

It has been recognised for some time that resection of liver metastases offers the best long-term hope for patients with mCRC. Chemotherapy regimens combining 5-FU/FA with irinotecan and/or oxaliplatin have demonstrated impressive efficacy in the downstaging of liver metastases to permit secondary resection (Van Cutsem *et al*, 2005) and their role in the neoadjuvant setting is now essentially undisputed. However, as yet, there is no consensus about which neoadjuvant regimen will be the most effective, either in general terms or for particular patients (as defined by disease or genotypic variation). Emerging data suggest that the addition of targeted biological agents to such regimens will further improve efficacy. Indeed, Folprecht *et al* (2006) reported a median survival time of 33 months, using cetuximab, an EGFR-targeted monoclonal antibody, in combination with irinotecan/5-FU/FA. In a randomised phase III trial, cetuximab was shown to increase response rate, progression-free survival and resection rate over FOLFIRI alone in patients with advanced CRC, with the PFS benefit being greatest for those patients with liver-only metastases (Van Cutsem *et al*, 2007).

One of the factors that have hindered comparisons between studies of neoadjuvant treatment of initially unresectable colorectal liver metastases is that there is no universally accepted definition of unresectability. Patient selection is a crucial factor, both for maximising the benefits achievable from primary resection (Fong *et al*, 1999) and for enabling the highest resection rates following neoadjuvant therapy (Adam *et al*, 2004a, 2004b; Folprecht *et al*, 2005). In their retrospective analysis, Folprecht *et al* (2005) found that in studies which enrolled patients with metastases confined to the liver, 24–54% of patients were able to undergo resection of their metastases subsequent to chemotherapy, whereas only 1–26% of patients in non-selective trials (patients with hepatic or extrahepatic disease) became eligible for secondary surgery. However, this analysis found a strong correlation between the response rate to neoadjuvant therapy and the resection rate in both the selected (*r*=0.96, *P*=0.002) and unselected (*r*=0.74, *P*<0.001) patients.

Studies of resection following neoadjuvant therapy have also demonstrated long-term benefits. Giacchetti *et al* (1999) reported that 77 out of 151 (51%) patients had surgery with curative intent following neoadjuvant treatment with 5-FU/FA/oxaliplatin. Complete resection was achieved in 58 patients. The median survival of the operated patients was 48 months (range: 25–71), and the 5-year survival rate was 50% (range: 38–61). A more recent retrospective analysis by Zaniboni *et al* (2005) of oxaliplatin-based chemotherapy found an overall response rate of 78.5% in 105 patients. Ninety-nine patients had liver surgery, with 79% undergoing radical resection. The median survival time for these patients was 42 months.

In a study using a neoadjuvant combination of 5-FU/FA/irinotecan/oxaliplatin (FOLFOXIRI) (Masi *et al*, 2006), there was a response rate of 72% in unselected patients and a 26% (19 patients) hepatic resection rate. In five patients, extrahepatic disease was also resected. The median overall survival of surgical patients was 36.8 months, with a 4-year survival rate of 37%. The overall survival of patients who responded to chemotherapy but did not undergo surgery was 22.2 months (*P*=0.0114). In a randomised phase III trial, FOLFOXIRI improved response rate, progression-free and overall survival compared with FOLFIRI, with secondary R0 resection rates of 15 *vs* 6% in non-selected patients and 36 *vs* 12% in patients with metastases confined to the liver (Falcone *et al*, 2007).

A multicentre phase II study investigated the use of irinotecan with 5-FU/FA (FOLFIRI) as neoadjuvant chemotherapy in selected patients with unresectable liver metastases (Ho *et al*, 2005). The overall response rate was 55% (22 out of 40 patients: 95% CI: 39.5–70.4%). Median overall survival was 20 months (95% CI: 17.7–26.6). Four patients underwent resection of metastases and remained alive at a median follow-up time of 33 months.

In the current study, neoadjuvant treatment with FOLFIRI achieved a response rate of 47.5%, with one-third of patients able to undergo secondary resection of liver metastases. Following resection, patients achieved a worthwhile median DF survival of 52.5 months. Median survival (all patients) was 31.5 months after a median follow-up of 56.0 months, and will be higher in radically resected patients, the median survival not yet having been reached. This compares favourably with Ho *et al* (2005) and other phase II studies in patients with initially unresectable metastases confined to the liver (Folprecht *et al*, 2005) and confirms the benefit of perioperative chemotherapy demonstrated in the EORTC EPOC trial in patients with resectable liver metastases (Nordlinger *et al*, 2007). Furthermore, the median overall survival is approaching twice that for unselected, unresected patients reported for combination therapy in the FOCUS (Seymour *et al*, 2007) and CAIRO trials (Punt *et al*, 2007).

As the use of neoadjuvant therapy has increased, so has the concern about possible long-term adverse effects of chemotherapy, particularly on the liver, where there is some evidence of vascular changes (Rubbia-Brandt *et al*, 2004; Adam *et al*, 2005) and steatohepatitis (Fernandez *et al*, 2005) following chemotherapy. However, although the vascular changes may increase the risk of intraoperative bleeding, it is not thought that they have any significant clinical impact (Adam *et al*, 2005).

Also, a retrospective study by Karoui *et al* (2006) showed that although preoperative chemotherapy was significantly associated with sinusoidal dilatation, atrophy of hepatocytes and/or hepatocytic necrosis (49 *vs* 25%, *P*=0.005) and increased morbidity, it did not appear to be linked to increased postoperative mortality in those patients who were resected. It is now generally accepted that provided patients are not overtreated with chemotherapy prior to surgery, there is little evidence of increased mortality and morbidity.

Ideally, the overall survival benefit conferred by neoadjuvant treatment needs to be validated in a prospective randomised trial in the palliative setting. Such a trial, in the light of present knowledge, would be ethically unacceptable because it should include a no surgery arm in which patients would not undergo surgery, even if their liver metastases become resectable after chemotherapy. However, prolonged (5 years or more) follow-up of patients, with identification of the particular regimen used to facilitate the resection of liver metastases, may be all that is necessary to fully evaluate the benefits of the different neoadjuvant treatment choices.

What is clear from the results of the current study is that irinotecan combination therapy (FOLFIRI) should be considered for the neoadjuvant treatment of patients with mCRC who have unresectable liver metastases as part of current strategic patient management practices. Future large-scale randomised studies in this setting should therefore include, for comparison, arms that receive irinotecan-based chemotherapy. Additionally, conventional chemotherapy could be combined with one or more molecularly targeted drugs as soon as the data on their role in advanced disease become available (Van Cutsem *et al*, 2007).

## Figures and Tables

**Table 1:** Baseline characteristics of patients

**Characteristic**	***n*=40 patients**
*Gender* (%)	
Male	60
Female	40
	
Median age, years (range)	58.7 (32–75)
	
*ECOG performance status* (%)	
0	97.5
1–2	2.5
	
*Metastases* (%)	
Synchronous	67.5
Metachronous	32.5
	
*Number of metastases* (%)	
<3	52.5
3–6	20
>6	27.5
	
*Largest metastasis* (%)	
⩾5 cm diameter	30
<5 cm diameter	70
	
*Site of metastasis* (%)	
Hilum	17.5
Other sites	82.5
	
*Main cause of unresectability* (%)	
Number of metastases	35
Location of metastases	35
Size of metastasis	25
Insufficient liver reserve	5

ECOG=Eastern Cooperative Oncology Group.

**Table 2:** Survival and time to progression in resected and non-resected patients

	**All patients *n*=40**	**Non-resected patients *n*=27**	**Resected patients *n*=13**
Median survival, months (range)	31.5 (4–92)	24	NR
Median TTP, months (range)	14.3 (2–49)	5.2 (2–13)	52.5
Median DF survival, months (range)	—	—	52.5 (2–89)
			
*Survival* (%)			
6 months	97.5	96.2	100 (85% DF)
1-year follow-up	87.5	81.4	100 (77% DF)
2-year follow-up	63.5	55.5	100 (62% DF)
Last follow-up (5 years)	22.5	3.7	62 (46% DF)
	(95% CI: 10.5, 34.5)		(OS 95% CI: 19.3, 72.9)
			(DF 95% CI: 19.3, 72.9)

CI=confidence interval; DF, disease-free; NR=not reached; OS=overall survival; TTP=time to progression.
